# Health-Related Quality of Life and Behavioral Difficulties in Greek Preschool Children with Developmental Language Disorder

**DOI:** 10.3390/healthcare12040470

**Published:** 2024-02-13

**Authors:** Konstantinos Kotsis, Maria Boukouvala, Alexandra Tzotzi, Iouliani Koullourou, Andromachi Mitropoulou, Aspasia Serdari, Vassiliki Siafaka, Thomas Hyphantis

**Affiliations:** 1 Department of Psychiatry, Faculty of Medicine, School of Health Sciences, University of Ioannina, 45 110 Ioannina, Greece; m.boukouvala@uoi.gr (M.B.); alexandratzotzi@gmail.com (A.T.); j.koulourou@uoi.gr (I.K.); mitropoulouandro@hotmail.com (A.M.); tyfantis@uoi.gr (T.H.); 2 Department of Child and Adolescent Psychiatry, Medical School, Democritus University of Thrace, 68 100 Alexandroupolis, Greece; aserntar@med.duth.gr; 3 Department of Speech & Language Therapy, School of Health Sciences, University of Ioannina, 45 500 Ioannina, Greece; siafaka@uoi.gr

**Keywords:** behavioral problems, developmental language disorder, emotional problems, health-related quality of life, kindergarten

## Abstract

Developmental language disorder (DLD) has a great impact on language skills as well as on a wide range of functioning areas, such as social and school functioning. In the present study, we aim to explore the Health-Related Quality of Life (HRQoL) of preschool children with DLD, compared to children with no language difficulties, using a self and proxy report method. A total of 230 parents of preschool children with DLD and 146 parents of children without language difficulties completed the Pediatric Quality of Life Inventory (PedsQL^TM^) 4.0 Generic Core Module and the Strengths and Difficulties Questionnaire (SDQ). Additionally, 71 children with DLD and 55 peers without DLD completed the self-reported PedsQL^TM^ module. The parents of kindergarten children (5–6 years old) with DLD reported that their kids experience worse social and school functioning compared to the control group. In addition, the children with DLD self-reported lower physical and social functioning. The parents of children with DLD reported that their children experience higher hyperactivity/inattention problems than the parents of the control group. Kindergarten children with DLD have a poorer HRQoL compared to their peers, as perceived by themselves and their parents. Moreover, children with DLD present with higher hyperactivity and inattention symptoms. Health professionals working with children who have DLD need to consider not only the language difficulties but also the children’s wellbeing and symptoms of hyperactivity and inattention.

## 1. Introduction

Developmental language disorder (DLD, formerly known as specific language impairment, as well as other terms) refers to low language abilities in the absence of biomedical conditions that could explain the deficit [1,2], and it is a quite common developmental disorder diagnosed in preschool years. Its prevalence is estimated to be approximately 7% in 5-year-old children [3]. DLD is a highly heterogeneous condition [1]. The main diagnostic features of DLD include difficulties in the acquisition, production, and use of language. Clinical manifestations include difficulties in expressive skills (less vocabulary than expected, shorter sentences with grammatical errors, and pronunciation) and/or in receptive abilities such as being able to decode information on sound, word, sentence, and text levels (e.g., word-finding problems, poor understanding of synonyms) [4,5]. The language abilities of people with DLD are below what would be expected for their age. Specifiers in ICD-11 include impairments in both expressive and receptive skills, mainly expressive skills, mainly pragmatic skills, and other impairments [6]. On the other hand, DSM-5 [5] does not have specifiers but, in the criterion A for its diagnosis, distinguishes deficits in comprehension or production. 

DLD has immediate and long-term impacts in children’s lives, and much of its impact extends beyond language difficulties. The literature [7,8,9,10] suggests that children with DLD are at an increased risk for poorer outcomes in social, emotional, behavioral, and academic functioning. Due to its high prevalence and life-long consequences, it is important for health professionals to understand the impact of DLD and employ measures to assess the Heath-Related Quality of Life (HRQoL) of people with this condition. This becomes even more important as DLD is considered an unknown disorder with hidden impairment [11]. HRQoL is the perception of the impact that a health condition has on one’s life [12]. It is a multidimensional construct that describes physical, social, and psychological aspects of wellbeing and functioning [13]. Over the last few decades it has become clear that HRQoL represents an important outcome that can guide decisions on interventions at a patient group level [14]. HRQoL has been explored in many childhood conditions, and early data were provided by caregivers [13]. However, children and caregivers do not necessarily share similar views about the impact of a health condition. Moreover, it is widely accepted that it is optimal for children to express their views freely in all matters affecting them and report their own perceptions for their HRQoL. Even if there are reports in the literature that children below the age of 7–8 years old have difficulties answering abstract topics, it is found that, when instructions are provided with age-appropriate information, children can communicate their health needs [15,16,17,18]. However, it is important, when we investigate difficulties in children, to also explore parental perceptions since they can make important contributions to our holistic understanding of the impact of a condition on a child and inform interventions. Taking that in account, HRQoL measurement tools assess the individual’s or a proxy’s perspective on the former’s wellbeing and usually incorporate physical, psychosocial, and social functioning [8]. 

Regarding DLD and HRQoL, a longitudinal study in Australia [8] found that children with DLD had a lower parent-reported QoL at the age of 9, compared to their peers without DLD. Moreover, a decline in the parent-reported QoL was recorded in children with DLD between the ages of 4 and 9, compared to their peers. For all children in this last study [8], their language skills at 7 years predicted their QoL at 9 years of age. Similar results were reported in a previous study [19], where children with DLD had significantly lower parent-reported HRQOL scores compared to children without DLD. A longitudinal analysis has shown that speech and language difficulties are associated with a reduced HRQoL, while typical language skills constitute a protective factor, positively associated with the HRQoL [20]. A recent systematic review revealed that all areas of functioning (e.g., physical, psychological, parent-relation, school environment, autonomy, positive moods) seem to be affected, with social functioning being the area most impaired [21]. Moreover, findings from the literature suggest that language skills at a preschool age predict social skills at later ages but not peer problems [22,23]. However, there are also reports that peer difficulties may arise as children grow older [24,25]. Preschool children with DLD may find everyday social communication hard both at home and in school based on their parents’ and teachers’ perspectives. Additionally, children with more severe presentations (expressive and receptive difficulties) face significantly more limitations in their communication [26]. Severity accounts for school functioning, as it has been found to be affected in children with severe DLD [8,19]. This highlights the importance of considering the impact of DLD on a child’s everyday life by measuring their HRQoL [27]. To the best of our knowledge, the literature is sparse regarding the HRQoL of preschool children with DLD, including both their own perspective and their parents. Therefore, it is important to focus on the preschool age by assessing both perspectives. Moreover, a comparison peers without DLD will provide a clearer picture about the impact of DLD on the HRQoL. 

The current study aims to fill this gap by using a parent-proxy and a self-report approach for assessing the HRQoL and investigating the following research questions:

(a) Do the parents of preschool children with DLD perceive more limitations of their children compared to the parents of peers without DLD?
(b) Do preschool children with DLD experience limitations in their self-reported HRQoL compared to their peers without DLD?

Our hypotheses are that both parents of children with DLD and children with DLD will score lower than their peers without DLD in all domains of functioning and, mainly, in the social one. 

Apart from the impact of DLD on one’s HRQoL, it is known that DLD has been associated with increased rates of problem behaviors, either internalizing or externalizing ones, such as attention deficit and hyperactivity symptoms [28,29,30]. It is also reported that symptoms of DLD and ADHD are frequently co-occurrent [30,31,32,33]. A study found that half of the children with language impairment display behavior problems independently of their age [34]. Older studies have also revealed similar results of high rates (>30% of the sample) of behavioral problems [35]. Moreover, a recent metanalysis reported that children with language disorders show greater rates of behavioral problems compared to their peers without DLD, and this difference is more pronounced in older children [36]. Furthermore, in a longitudinal study [8], emotional symptoms and peer problems at 4 years of age in children with DLD were predictive of lower HRQoL rates at 9 years of age. Therefore, as a secondary aim, we also explore the following research question:

(c) do the parents of preschool children with DLD perceive more behavioral and emotional problems in their children compared to the parents of peers without DLD?

Similar to the HRQoL, our hypothesis is that the parents of children with DLD will score lower in emotional and behavioral problems than the parents of children without DLD. 

## 2. Materials and Methods

### 2.1. Participants

The participants included 230 Greek-speaking parents of preschool (90 nursery and 140 kindergarten) children with DLD and 146 parents (44 nursery and 102 kindergarten) of children with no reported language difficulties. Moreover, the sample of children consisted of 71 children with DLD and 55 peers without DLD. The mean age of the parents of children with DLD was 39.1 ± 5.6 and that of the parents of children without language problems was 36.6 ± 3.9. The mean age of the children was 4.7 ± 1.0 and 4.5 ± 0.8, respectively. No difference in children’s age was noted (*p* = 0.545); however, the parents of children with DLD were older (*p* < 0.001). In Greece, children attend nursery school until they reach the age of 4 years, and then they attend kindergarten (mandatory education) for two years (5–6 years old). 

### 2.2. Measures

In this study, the Pediatric Quality of Life Inventory (PedsQL^TM)^ 4.0 Generic Core Module assessing self- and parent-proxy-reported HRQoL was used [37,38,39,40]. The Greek version demonstrated suitability for application in school and community healthcare settings [41]. Specifically, the young child self-report and parent-proxy version for ages 5–7 and the Parent Report for Toddlers 2–4 were used. The young child self-report version employs a simplified three-point Likert scale going from “not at all” to “a lot” with smiley faces to assist children below 7 years of age in completing the scale. The parent-proxy version employs a five-point Likert scale going from “never” (indicated as zero) to “almost always” (indicated as four). Both versions consisted of twenty-three items in four dimensions: physical functioning (eight items), emotional functioning (five items), social functioning (five items), and school functioning (five items). Each statement asks the child or the parent the following question: “How much of a problem has this been for you/your child?”. Physical functioning refers to problems such as walking, running, low energy; emotional functioning refers to problems with emotions such as feeling scared or afraid, sleep problems, and anxiety; social functioning refers to problems with peers such as getting along with other children, being teased by other children, and not being able to keep up when playing with other children; school functioning refers to problems in school such as paying attention, missing school, and having problems with keeping up with schoolwork. The scales produce two summary scores: a psychosocial health summary (sum of the emotional, social, and school functioning dimensions) and a physical health summary score, which is identical to the physical functioning dimension. Finally, a total score is calculated from the sum of all the items over the number of items answered (this accounts for missing data). The items within a dimension are reverse-scored and linearly transformed to a 0–100 scale (e.g., 0 = 100, 1 = 75, 2 = 50, 3 = 15, 4 = 0), with higher scores indicating a better HRQoL. If more than 50% of the items in the scale are missing, the scale score is not computed. The reliability of the scale utilized in this study was good based on McDonald’s omega for all versions: parent-proxy version = 0.89, young child self-report = 0.76, and parent-report for toddlers = 0.83.

Strengths and Difficulties Questionnaire (SDQ—Parent Version): The SDQ [42,43] is a brief behavioral screening questionnaire completed by the parents of 4–17-year-old children. The SDQ records 25 attributes that are divided into the following five subscales: (a) emotional symptoms such as worries, unhappiness, nervousness, and somatic complaints; (b) conduct problems such as temper tantrums, obedience, and cheating; (c) hyperactivity/inattention such as being restless, overactive, fidgeting, and distracting; (d) peer relationship problems such as being bullied, playing alone, and having one friend; and (e) prosocial behavior such as considering other people’s feelings, being helpful if someone is hurt, and being kind to younger children. The responses to each of the 25 items consist of three options: not true, somewhat true, or certainly true. The first four subscales are added together to generate a total difficulties’ score. High scores on the four subscales and the total difficulties scale represent a high degree of difficulties; a high score on the prosocial subscale represents a high degree of prosocial behavior. The reliability of the scale utilized in our study was low based on McDonald’s omega (ω = 0.59).

### 2.3. Procedure

Data were collected during the assessment of a requested evaluation for children language problems to a Community Child and Adolescent Mental Health Service (CAMHS) belonging to a tertiary University Hospital. The data collection represents part of an on-going project that aims to explore factors associated with HRQoL in preschool children with DLD. All the children diagnosed with DLD, according to the ICD criteria in our CAMHS, and their parents were asked to participate in this study. None of the parents or children refused to participate in this study. Parents of children with no reported language difficulties were recruited from nursery and kindergarten schools after permission from the Municipality and the Regional Education Directorate, respectively. Moreover, children with DLD were recruited from the same CAMHS and their peers without DLD from the same kindergarten schools as their parents, respectively. The kindergarten children and their parents were asked to complete the questionnaires. The toddlers from the nursery schools did not complete any questionnaire. 

All the children with DLD in our sample underwent clinical assessment by a speech language therapist. A psychiatric evaluation was carried out to rule out major develop-mental disorders such as autism spectrum disorder (ASD) or intellectual disability (ID). The eligibility criteria were the following: (a) being diagnosed with a developmental language disorder; (b) not following a speech—language intervention; (c) being native Greek speakers; (d) not showing receptive language difficulties; and (e) not suffering from any medical condition or having any mental or other developmental disorders. 

The steps for data collection in the DLD group by completing the paper-and-pencil questionnaire were as follows: all the parents and children were informed about this study’s objectives and confidentiality by the speech and language therapist. The parents provided written consent while the children gave their verbal consent. The parents completed the parental forms in the waiting room of the CAMHS, while the children independently filled out the questionnaire administered by the speech and language therapist after their child psychiatry evaluation, without the presence of their parents. The procedure for data collection among the control group was the following: directors of nursery and kindergarten schools were contacted by the researchers in person, and they were informed about this study. Subsequently, parents were informed about the aim of this study either by the school directors or in groups by the researchers, while providing a psychoeducational lecture about typical language development in a preschool age. The parents agreed to participate, signed the consent forms, completed the proxy report (paper-and-pencil questionnaires) at home (their kindergarten children too) and submitted them to the school in a sealed envelope. The directors of the schools then notified the main researcher to collect the questionnaires. 

This study was conducted according to the guidelines of the Declaration of Helsinki, and ethical approval was obtained from the Ethical Committee of our institution (University Hospital of Ioannina, Reference number: 989—21 December 2020).

### 2.4. Design and Statistical Analysis

Our study is a comparative cross-sectional natural group design, and, considering this, we compared the mean values that children and parents recorded in the variables of each instrument. A Kolmogorov–Smirnov test for all the variables rejected the null hypothesis (*p <* 0.001), revealing that the variables in our study do not follow a normal distribution. Therefore, the non-parametric Mann–Whitney U test was used to compare the PedsQL and SDQ mean scores of all the variables between the parents of children with DLD and the parents of the control group for toddlers and kindergarten children separately. The same statistical test was used to compare the PedsQL mean scores of all the variables between the kindergarten children with DLD and the kindergarten children with typical language development. For the statistical analysis, the Statistical Package for Social Sciences v28 for MacOS was used.

## 3. Results

### 3.1. Parent-Reported HRQoL 

Table 1 shows that the parents of nursery-aged children reported that the best HRQoL domain of functioning was the physical one, and the worst domain was the emotional one, for both the DLD group and the control group. No statistically significant differences were observed in any HRQoL functioning domain, meaning that the parents of nursery-aged children with DLD did not report a different HRQoL to the parents of children without language difficulties. The same pattern in terms of the best and the worst HRQoL domains of functioning was observed in the kindergarten children; the physical domain of functioning was the one with the highest score and the emotional one was the domain with the lowest. However, in that age group, the parents of the control group reported statistically significant better HRQoL scores in the social (*p* = 0.043, r = 0.130) and school (*p* = 0.009, r = 0.167) domains of functioning compared to the parents of children with DLD. These results indicate that children with DLD have more difficulties with their peers and face more challenges at school. 

### 3.2. Children Self-Reported HRQoL

The children’s rating of their own HRQoL showed differences between those with DLD and those without language problems (Table 2). Specifically, the children with DLD scored statistically significantly lower (indicating a worse HRQoL) in the physical *p* = 0.02, r = 0.195) and social (*p* = 0.04, r = 0.176) dimensions of the PedsQL. Consequently, the psychosocial summary score (*p* = 0.05, r = 0.173) and the total score (*p* = 0.03, r = 0.189) of the PedsQL were lower in the DLD group, indicating a self-reported worse HRQoL for the children with DLD. 

### 3.3. Behavioral and Emotional Problems

The results (Table 3) did not indicate significant differences between the DLD group and the control group in emotional and behavioral problems, except for hyperactivity/inattention. Specifically, the parents of children with DLD reported statistically significant higher (*p* < 0.001, r = 0.253) hyperactivity/inattention problems than the parents of the control group’s children. 

## 4. Discussion

This study aimed to explore the HRQoL of preschool children with DLD compared to their peers without language problems both from the parents’ and the children’ perspective. A secondary aim was to explore whether preschool children with DLD differ in terms of behavioral and emotional difficulties from their peers. 

As it might be expected, the parents of the control group recorded higher scores and, therefore, perceived their children’s functioning to be better than that recorded by the parents of the DLD group. Considering the different nature of nursery and kindergarten schools, our results are different for these age groups. Regarding nursery schools, no statistical differences were revealed in any aspect of functioning. This might be explained by the fact that, in nursery school, social activities are mainly guided and supported by the teachers, considering the very young age of the children. Furthermore, there are no homework or school exercises, and the PedsQL for children who are 2–4 years old asks mainly about school absences, which do not constitute a usual consequence of DLD. However, the results were not similar for kindergarten children. Social and school functioning were perceived by the parents of children with DLD to be poorer compared to the results reported by the parents of the control group. This is not a surprising finding and reflects the nature of the condition itself, considering that DLD refers to children who have “*language problems enduring into middle childhood and beyond, with a significant impact on everyday social interactions or educational progress” and that “lead to significant functional impairments unlikely to resolve without specialist help*” [1] *(p. 1070).*

The negative association of social functioning and communication impairments is a consistent finding in the literature, with small variations depending on the children’s age and the instruments used in the studies [21,44,45]. Considering the studies which used PedsQL, a recent study [8] found that a low language was associated with all the HRQoL domains of functioning for children younger than 9 years of age. A very recent study [26] found that children who are 5–6 years old with DLD (receptive–expressive) face significant challenges in a variety of communication situations. Specifically, their schoolteachers rated their communicative abilities as inadequate; however, their social competence was rated as normal. Preschoolers’ language skills are moderate predictors for social skills at the age of 8 years and for language skills at age of 7 years and do not significantly predict peer problems at ages 7 or 11 in children with DLD [22,23]. Yet, when children with DLD grow older, they experience increasing levels of peer difficulties even if there is evidence that their communicative skills increase from a preschool age to a school age [24,25,46]. In our study, even at that young age, parents tended to perceive that the social skills of their children were poorer compared with those of their peers. 

Additionally, the parents of kindergarten children with DLD in our study reported a lower school functioning than the parents of the control group. This result is also consistent with the international literature regarding the fact that children with DLD face significant challenges in school, since it is well-known that they need adequate language skills to engage in school learning [8,19,30,47]. In a longitudinal study, children with severe DLD scored significantly lower on the PedsQL school functioning scale at 9 years of age [8]. Several studies revealed similar results: a poorer school functioning of children with DLD compared to their peers without DLD [19,45]. However, the samples of these studies consisted mainly of school-aged children. Interestingly, in our study, the parents of kindergarten children with DLD noticed school functioning challenges compared to their peers without DLD. This seems a rather important finding in terms of prevention, indicating that children with DLD should be monitored from preschool regarding their school functioning and should be offered language interventions. In Greece, speech–language interventions are not offered in the school setting but are mainly offered in the private sector and, less frequently, in the public sector, mainly during school and working hours.

While differences were reported by the parents of children with DLD and those of the control group in social and school functioning, the mean scores were in the normal range compared to the mean scores published by Varni et al. [40]. Any score below the mean scores published by Varni indicates groups “at-risk” for an impaired HRQoL. Considering this, both of our groups did not represent high-risk groups; however, we should take into account the fact that the scores of Varni’s study may vary across countries. The same occurs when considering the Greek mean scores published by Gkoltsiou et al.; however, the sample size in their study consisted of parents of children over 8 years of age [41]. 

This study additionally aimed to compare the HRQoL perceived by the children themselves, and, to the best of our knowledge, this is the first study including solely children of this age group. The self-perceived HRQOL of the children with DLD in our study was significantly lower in the physical and social dimensions and, subsequently, in the psychosocial summary score and in the total score compared to the control group. The findings on physical functioning were rather unexpected, since the main challenge of these children was communication. However, this could be explained by the comorbidity of motor difficulties in children with speech–language difficulties that the children in our sample might have had. The literature suggests that some motor difficulties co-occur in up to 90% of children with speech–language difficulties, while developmental coordination disorders may be found in 32.3% of children with a specific language impairment (the former nomenclature of DLD) [48,49,50]. Concerning the finding that the children with DLD in our study reported significantly poorer social functioning compared to their peers without DLD, this might have been due to a direct impact of their language skills. This is a well-known issue in the literature, where children with language problems may find it difficult to initiate interactions with peers, successfully participate in ongoing interactions, and get along with their peers [51,52,53,54]. In a study [55] using self-reported questionnaires, children with DLD scored lower in conflict resolution and negotiation scenarios, requiring an appropriate use of language in complex contextual circumstances. The authors of the above-mentioned study hypothesized that children with DLD reacted to communicative interactions with socially inaccurate verbal and non-verbal responses.

Considering the self- and proxy reports, our study revealed some interesting similarities and differences of the DLD group compared to the control group. Firstly, both the parents and children of the DLD group scored significantly lower in social functioning compared to the control group. It seems clear that children with DLD have difficulties in their social interactions. Children might be aware of their challenging social interactions at school and other settings and parents might notice challenges in the out-of-school social activities where they accompany their children due to their young age. On the other hand, the parents of the DLD group in our study reported a poorer school functioning than the parents of the control group, whereas this was not the case in the self-reported measure. This could be explained by the young age of the children; it might be difficult for preschoolers to clearly notice if they are facing school and mainly learning difficulties, since the basic aim of kindergarten in Greece is social and emotional development [56]. Furthermore, the parents of the DLD group failed to notice deficits in physical functioning compared to the parents of the control group, whereas the children acknowledge some physical challenges compared to their peers without DLD. Parents may not accurately identify physical challenges, especially if their children are not physically active (e.g., sport activities), and may not be fully aware of the motor developmental milestones at that age. On the other hand, it is well known that children can more easily answer questions regarding their physical health and observable domains (compared to mental health and abstract domains) and, therefore, might record even minor physical challenges in their functioning [15,27] compared to their peers. Differences, although in terms of disagreement, were found in a previous study of our group with part of the same dataset consisting only of mothers; children with DLD and their mothers showed very poor agreement in all aspects of HRQoL [57]. This finding, together with those of the present study, further supports that children and parents may have different perspectives of HRQoL and highlights the importance of considering both perspectives when assessing children with DLD. 

Overall, similar findings have been revealed from qualitative studies as well; children with low language have difficulties in forming and maintaining peer relationships, completing tasks independently, and making their own decisions at school [18,58]. 

A secondary aim of this study was to explore whether parents of the DLD group reported differences in externalizing and internalizing symptoms compared to the parents of the control group. Our results showed that the parents of the DLD group reported significantly higher levels of hyperactivity/inattention than the parents of children without DLD. This is in line with the literature, where 20–30% of children with language impairments also have ADHD and display greater rates of problem behaviors compared to their peers without DLD [33,36,59]. On the other hand, as many as 50% of children with ADHD have language difficulties, and the overlap in symptomatology makes it difficult to differentiate these two conditions, especially at a preschool age [33]. However, much of the research conducted on these disorders has focused on school-aged children. Our findings highlight the importance of investigating symptoms of ADHD in preschool children with DLD. 

### Strengths and Limitations

Our study is not without limitations, and its findings should be interpreted with caution. Firstly, we did not account for DLD severity; therefore, we cannot argue that differences compared to peers without DLD may apply to various severity presentations. Studies with different severities may help clarify this possible association. Second, the lack of cutoff scores for an impaired HRQoL in a preschool age in Greece does not allow us to make assumption about the impairment of our DLD sample. Third, the DLD group and the control group in our study were recruited in different periods; the DLD group was recruited during the COVID era and the control group after the lifting of restrictive measures. Therefore, we cannot exclude the possibility that COVID had an impact on the HRQoL of the children and their parents. Additionally, the use of a generic HRQoL measure instead of a DLD-specific one might have resulted in missing important dimensions of functioning specific to children with DLD. Moreover, the lack of teacher ratings limits the interpretation of the findings related to school functioning. Future research should include teachers as informants. Furthermore, the kindergarten children with DLD completed their questionnaires with the presence of a speech–language therapist, while the control group completed them in their home, together with their parents; therefore, the different settings might have influenced the results. Also, the lack of standardized assessment measures (for the DLD diagnosis as well as the exclusion conditions) represents another limitation of our study. Finally, the results of this study represent a secondary analysis to our ongoing project exploring factors associated with HRQoL in preschool children with DLD. 

However, the sample size, the control group, the focus on a preschool age, the self-reported perception of HRQoL at that age, and the clinical assessment carried out represent the strengths of our study. 

Future studies should follow a longitudinal design to identify the impact of DLD over the years and the role of early interventions. Of significant importance is the identification of which features of these interventions could facilitate improvements in all aspects of HRQoL. Longitudinal studies will also give the opportunity to identify predictors of various other outcomes, such as externalizing or internalizing problems. Moreover, it is important to construct tools specific to DLD to catch all information and clinical variables related to it and measure severity. 

## 5. Conclusions

The parents of children with DLD and the children with DLD themselves acknowledged challenges (lower scores) in social, school, and physical aspects of HRQoL compared to the parents and children of the control group, respectively. Healthcare professionals working with children with DLD should assess not only language skills but also various domains of functioning to suggest focused interventions. Prioritizing HRQoL assessments will facilitate the care of DLD from a biopsychosocial perspective. Moreover, children with DLD should be helped to engage more in social and physical activities, and their parents and teachers should follow up more closely their academic functioning. Social difficulties in school might serve as an alert for referral and early diagnosis, whereas, in children diagnosed with DLD, teachers should promote further social interactions. In general, since social and school functioning is affected in this condition, an integrative effort involving education, health services, and community groups (e.g., sports activities) is needed to address the needs of children with DLD. Furthermore, clinicians assessing children with DLD should also assess symptoms of hyperactivity and inattention. Finally, children with DLD should be given the opportunity to express their own perspective along with that expressed by their parents.

## Figures and Tables

**Table 1:** Parent-reported HRQoL functioning for children with DLD and the control group for both age ranges (toddlers and kindergarten children).

PedsQL Dimension	Toddlers (2–4 Years Old)				Kindergarten (5–6 Years Old)			
	DLD (M ± SD)	Control (M ± SD)	*p*	ES r	DLD (M ± SD)	Control (M ± SD)	*p*	ES r
Physical	93.08 ± 7.83	92.97 ± 8.06	0.929	0.001	85.1 ± 18.74	85.36 ± 12.9	0.208	0.081
Emotional	82.25 ± 17.33	83.30 ± 16.13	0.843	0.002	83.77 ± 14.81	84.36 ± 14.25	0.864	0.011
Social	86.97 ± 15.12	90.91 ± 11.82	0.143	0.127	83.59 ± 17.94	88.63 ± 13.44	0.043 *	0.130
School	89.89 ± 14.29	86.17 ± 15.96	0.192	0.113	83.66 ± 16.05	88.97 ± 13.54	0.009 **	0.167
Psychosocial Summary	85.83 ± 12.46	86.89 ± 10.97	0.844	0.002	83.67 ± 12.67	87.32 ± 11.08	0.023 *	0.147
Total score	88.59 ± 9.34	89.20 ± 8.60	0.850	0.016	84.17 ± 13.25	86.64 ± 10.91	0.263	0.072

* significant at the 0.05 level, ** significant at the 0.01 level.

**Table 2:** Kindergarten children’s self-reported HRQoL functioning for children with DLD and the control group.

PedsQL Dimension		Kindergarten Children (5–6 Years Old)		
	DLD (M ± SD)	Control (M ± SD)	*p*	ES r
Physical	71.83 ± 12.93	77.16 ± 14.59	0.02 *	0.195
Emotional	66.48 ± 20.36	71.45 ± 21.29	0.16	0.125
Social	71.41 ± 20.58	78.73 ± 17.21	0.04 *	0.176
School	75.21 ± 16.02	77.45 ± 16.24	0.42	0.070
Psychosocial Summary	71.03 ± 14.13	75.58 ± 15.38	0.05 *	0.173
Total score	71.31 ± 12.20	76.13 ± 13.26	0.03 *	0.189

* significant at the 0.05 level.

**Table 3:** Parent reports on the emotional and behavioral problems of their children.

SDQ Subscales				
	DLD (M ± SD)	Control (M ± SD)	*p*	ES r
Emotional symptoms	1.27 ± 1.61	1.23 ± 1.45	0.90	0.006
Conduct problems	1.87 ± 1.78	1.46 ± 1.37	0.89	0.091
Hyperactivity/inattention	2.95 ± 1.96	1.93 ± 1.58	<0.001 ***	0.253
Peer relationship problems	1.01 ± 1.27	0.95 ± 1.21	0.98	0.023
Prosocial behavior	8.06 ± 1.59	8.23 ± 1.80	0.85	0.086
Total score	7.10 ± 4.62	5.57 ± 3.91	0.103	0.162

*** significant at the 0.001 level.

## Data Availability

Data are available from the authors upon request.

## References

[1] Bishop D.V.M., Snowling M.J., Thompson P.A., Greenhalgh T. (2017). Phase 2 of CATALISE: A Multinational and Multidisciplinary Delphi Consensus Study of Problems with Language Development: Terminology. J. Child Psychol. Psychiatry.

[2] Bishop D.V.M., Snowling M.J., Thompson P.A., Greenhalgh T. (2016). CATALISE: A Multinational and Multidisciplinary Delphi Consensus Study. Identifying Language Impairments in Children. PLoS ONE.

[3] Norbury C.F., Gooch D., Wray C., Baird G., Charman T., Simonoff E., Vamvakas G., Pickles A. (2016). The Impact of Nonverbal Ability on Prevalence and Clinical Presentation of Language Disorder: Evidence from a Population Study. J. Child Psychol. Psychiatry.

[4] Lisa R., Pola R., Franz P., Jessica M. (2019). Developmental Language Disorder: Maternal Stress Level and Behavioural Difficulties of Children with Expressive and Mixed Receptive-Expressive DLD. J. Commun. Disord..

[5] American Psychiatric Association (2013). Diagnostic and Statistical Manual of Mental Disorders.

[6] World Health Organization (2021). International Statistical Classification of Diseases and Related Health Problems.

[7] Bretherton L., Prior M., Bavin E., Cini E., Eadie P., Reilly S. (2014). Developing Relationships between Language and Behaviour in Preschool Children from the Early Language in Victoria Study: Implications for Intervention. Emot. Behav. Diffic..

[8] Eadie P., Conway L., Hallenstein B., Mensah F., McKean C., Reilly S. (2018). Quality of Life in Children with Developmental Language Disorder. Int. J. Lang. Commun. Disord..

[9] Nicola K., Watter P. (2018). The Comparison of Perceived Health-Related Quality of Life between Australian Children with Severe Specific Language Impairment to Age and Gender-Matched Peers. BMC Pediatr..

[10] Lyons R. (2021). Impact of Language Disorders on Children’s Everyday Lives from 4 to 13 Years: Commentary on Le, Mensah, Eadie, McKean, Schiberras, Bavin, Reilly and Gold (2020). J. Child Psychol. Psychiatry.

[11] McGregor K.K. (2020). How We Fail Children with Developmental Language Disorder. Lang. Speech Hear. Serv. Sch..

[12] Spieth L.E., Harris C.V. (1996). Assessment of Health-Related Quality of Life in Children and Adolescents: An Integrative Review. J. Pediatr. Psychol..

[13] Orbell S., Schneider H., Esbitt S., Gonzalez J.S., Gonzalez J.S., Shreck E., Batchelder A., Gidron Y., Pressman S.D., Hooker E.D. (2013). Health-Related Quality of Life. Encyclopedia of Behavioral Medicine.

[14] Koot H.M., Wallander J.L., Koot H.M. (2001). The Study of Quality of Life: Concepts and Methods. Quality of Life in Child and Adolescent Illness. Concepts, Methods and Findings.

[15] Coghill D., Danckaerts M., Sonuga-Barke E., Sergeant J. (2009). Practitioner Review: Quality of Life in Child Mental Health–Conceptual Challenges and Practical Choices. J. Child Psychol. Psychiatry.

[16] Brewster A.B. (1982). Chronically Ill Hospitalized Children’s Concepts of Their Illness. Pediatrics.

[17] Pantell R.H., Lewis C.C. (1987). Measuring the Impact of Medical Care on Children. J. Chronic Dis..

[18] Markham C., van Laar D., Gibbard D., Dean T. (2009). Children with Speech, Language and Communication Needs: Their Perceptions of Their Quality of Life. Int. J. Lang. Commun. Disord..

[19] Hubert-Dibon G., Bru M., Gras Le Guen C., Launay E., Roy A. (2016). Health-Related Quality of Life for Children and Adolescents with Specific Language Impairment: A Cohort Study by a Learning Disabilities Reference Center. PLoS ONE.

[20] Feeney R., Desha L., Khan A., Ziviani J. (2017). Contribution of Speech and Language Difficulties to Health-Related Quality-of-Life in Australian Children: A Longitudinal Analysis. Int. J. Speech Lang. Pathol..

[21] Le H.N.D., Le L.K.D., Nguyen P.K., Mudiyanselage S.B., Eadie P., Mensah F., Sciberras E., Gold L. (2020). Health-Related Quality of Life, Service Utilization and Costs of Low Language: A Systematic Review. Int. J. Lang. Commun. Disord..

[22] Mok P.L.H., Pickles A., Durkin K., Conti-Ramsden G. (2014). Longitudinal Trajectories of Peer Relations in Children with Specific Language Impairment. J. Child Psychol. Psychiatry.

[23] Aro T., Eklund K., Nurmi J.-E., Poikkeus A.-M. (2012). Early Language and Behavioral Regulation Skills as Predictors of Social Outcomes. J. Speech Lang. Hear. Res..

[24] Singer I., de Wit E., Gorter J.W., Luinge M., Gerrits E. (2023). A Systematic Scoping Review on Contextual Factors Associated with Communicative Participation among Children with Developmental Language Disorder. Int. J. Lang. Commun. Disord..

[25] Cunningham B.J., Hanna S.E., Oddson B., Thomas-Stonell N., Rosenbaum P. (2017). A Population-based Study of Communicative Participation in Preschool Children with Speech-language Impairments. Dev. Med. Child Neurol..

[26] Bruinsma G.I., Wijnen F., Gerrits E. (2023). Communication in Daily Life of Children with Developmental Language Disorder: Parents’ and Teachers’ Perspectives. Lang. Speech Hear. Serv. Sch..

[27] Ottosson S., Schachinger Lorentzon U., Kadesjö B., Gillberg C., Miniscalco C. (2022). Neurodevelopmental Problems and Quality of Life in 6-year-olds with a History of Developmental Language Disorder. Acta Paediatr..

[28] Van Daal J., Verhoeven L., Van Balkom H. (2007). Behaviour Problems in Children with Language Impairment. J. Child Psychol. Psychiatry.

[29] McCabe P.C. (2005). Social and Behavioral Correlates of Preschoolers with Specific Language Impairment. Psychol. Sch..

[30] McKean C., Reilly S., Bavin E.L., Bretherton L., Cini E., Conway L., Cook F., Eadie P., Prior M., Wake M. (2017). Language Outcomes at 7 Years: Early Predictors and Co-Occurring Difficulties. Pediatrics.

[31] Redmond S.M. (2016). Language Impairment in the Attention-Deficit/Hyperactivity Disorder Context. J. Speech Lang. Hear. Res..

[32] Baker L., Cantwell D.P. (1992). Attention Deficit Disorder and Speech/Language Disorders. Compr. Ment. Health Care.

[33] Parks K.M.A., Hannah K.E., Moreau C.N., Brainin L., Joanisse M.F. (2023). Language Abilities in Children and Adolescents with DLD and ADHD: A Scoping Review. J. Commun. Disord..

[34] Maggio V., Grañana N.E., Richaudeau A., Torres S., Giannotti A., Suburo A.M. (2014). Behavior Problems in Children With Specific Language Impairment. J. Child Neurol..

[35] Willinger U., Brunner E., Diendorfer-Radner G., Sams J., Sirsch U., Eisenwort B. (2003). Behaviour in Children with Language Development Disorders. Can. J. Psychiatry.

[36] Curtis P.R., Frey J.R., Watson C.D., Hampton L.H., Roberts M.Y. (2018). Language Disorders and Problem Behaviors: A Meta-Analysis. Pediatrics.

[37] Varni J.W., Seid M., Kurtin P.S. (2001). PedsQL^TM^ 4.0: Reliability and Validity of the Pediatric Quality of Life Inventory^TM^ Version 4.0 Generic Core Scales in Healthy and Patient Populations. Med. Care.

[38] Varni J.W., Limbers C.A., Burwinkle T.M. (2007). Parent Proxy-Report of Their Children’s Health-Related Quality of Life: An Analysis of 13,878 Parents’ Reliability and Validity across Age Subgroups Using the PedsQL^TM^ 4.0 Generic Core Scales. Health Qual. Life Outcomes.

[39] Varni J.W., Limbers C.A., Burwinkle T.M. (2007). How Young Can Children Reliably and Validly Self-Report Their Health-Related Quality of Life?: An Analysis of 8,591 Children across Age Subgroups with the PedsQL^TM^ 4.0 Generic Core Scales. Health Qual. Life Outcomes.

[40] Varni J.W., Burwinkle T.M., Seid M., Skarr D. (2003). The PedsQL^TM^* 4.0 as a Pediatric Population Health Measure: Feasibility, Reliability, and Validity. Ambul. Pediatr..

[41] Gkoltsiou K., Dimitrakaki C., Tzavara C., Papaevangelou V., Varni J.W., Tountas Y. (2008). Measuring Health-Related Quality of Life in Greek Children: Psychometric Properties of the Greek Version of the Pediatric Quality of Life Inventory^TM^ 4.0 Generic Core Scales. Qual. Life Res..

[42] Giannakopoulos G., Tzavara C., Dimitrakaki C., Kolaitis G., Rotsika V., Tountas Y. (2009). The Factor Structure of the Strengths and Difficulties Questionnaire (SDQ) in Greek Adolescents. Ann. Gen. Psychiatry.

[43] Goodman R. (1997). The Strengths and Difficulties Questionnaire: A Research Note. J. Child Psychol. Psychiatry.

[44] Charman T., Ricketts J., Dockrell J.E., Lindsay G., Palikara O. (2015). Emotional and Behavioural Problems in Children with Language Impairments and Children with Autism Spectrum Disorders. Int. J. Lang. Commun. Disord..

[45] Feeney R., Desha L., Ziviani J., Nicholson J.M. (2012). Health-Related Quality-of-Life of Children with Speech and Language Difficulties: A Review of the Literature. Int. J. Speech Lang. Pathol..

[46] Cunningham B.J., Hanna S.E., Rosenbaum P., Thomas-Stonell N., Oddson B. (2018). Factors Contributing to Preschoolers’ Communicative Participation Outcomes: Findings From a Population-Based Longitudinal Cohort Study in Ontario, Canada. Am. J. Speech Lang. Pathol..

[47] Snowling M.J., Duff F.J., Nash H.M., Hulme C. (2016). Language Profiles and Literacy Outcomes of Children with Resolving, Emerging, or Persisting Language Impairments. J. Child Psychol. Psychiatry.

[48] Hill E.L. (2001). Non-specific Nature of Specific Language Impairment: A Review of the Literature with Regard to Concomitant Motor Impairments. Int. J. Lang. Commun. Disord..

[49] Flapper B.C.T., Schoemaker M.M. (2013). Developmental Coordination Disorder in Children with Specific Language Impairment: Co-Morbidity and Impact on Quality of Life. Res. Dev. Disabil..

[50] Finlay J.C.S., McPhillips M. (2013). Comorbid Motor Deficits in a Clinical Sample of Children with Specific Language Impairment. Res. Dev. Disabil..

[51] Fujiki M., Brinton B., Todd C.M. (1996). Social Skills of Children With Specific Language Impairment. Lang. Speech Hear. Serv. Sch..

[52] Brinton B., Fujiki M., Spencer J.C., Robinson L.A. (1997). The Ability of Children With Specific Language Impairment to Access and Participate in an Ongoing Interaction. J. Speech Lang. Hear. Res..

[53] Brinton B., Fujiki M., McKee L. (1998). Negotiation Skills of Children With Specific Language Impairment. J. Speech Lang. Hear. Res..

[54] Craig H.K., Washington J.A. (1993). Access Behaviors of Children With Specific Language Impairment. J. Speech Lang. Hear. Res..

[55] Marton K., Abramoff B., Rosenzweig S. (2005). Social Cognition and Language in Children with Specific Language Impairment (SLI). J. Commun. Disord..

[56] Ralli A.M., Kalliontzi E., Kazali E. (2022). Teachers’ Views of Children With Developmental Language Disorder in Greek Mainstream Schools. Front. Educ..

[57] Boukouvala M., Hyphantis T., Koullourou I., Tzotzi A., Mitropoulou A., Mantas C., Petrikis P., Serdari A., Siafaka V., Kotsis K. (2023). Health-Related Quality of Life in Kindergarten Children with Developmental Language Disorder: Child–Mother Agreement. Behav. Sci..

[58] Markham C., Dean T. (2006). Parents’ and Professionals’ Perceptions of Quality of Life in Children with Speech and Language Difficulty. Int. J. Lang. Commun. Disord..

[59] Beitchman J., Tuckett M., Batth S. (1987). Language Delay and Hyperactivity in Preschoolers: Evidence for a Distinct Subgroup of Hyperactives. Can. J. Psychiatry.

